# Supplementary material for: Recurrent evolution of high virulence in isolated populations of a DNA virus
Source: eLife. 2020 Oct 28;9:e58931. doi: 10.7554/eLife.58931 (PMC7685711; doi:10.7554/eLife.58931)
Supplement: Transparent reporting form [file elife-58931-transrepform.docx]

***eLife’s* transparent reporting form**

We encourage authors to provide detailed information *within their submission* to facilitate the interpretation and replication of experiments. Authors can upload supporting documentation to indicate the use of appropriate reporting guidelines for health-related research (see [EQUATOR Network](http://www.equator-network.org/%20)), life science research (see the [BioSharing Information Resource](https://biosharing.org/)), or the [ARRIVE guidelines](http://www.plosbiology.org/article/info:doi/10.1371/journal.pbio.1000412) for reporting work involving animal research. Where applicable, authors should refer to any relevant reporting standards documents in this form.

If you have any questions, please consult our Journal Policies and/or contact us: [editorial@elifesciences.org](mailto:editorial@elifesciences.org).

**Sample-size estimation**

- You should state whether an appropriate sample size was computed when the study was being designed
- You should state the statistical method of sample size computation and any required assumptions
- If no explicit power analysis was used, you should describe how you decided what sample (replicate) size (number) to use

Please outline where this information can be found within the submission (e.g., sections or figure legends), or explain why this information doesn’t apply to your submission:

There are several experiments considered here. Generally speaking, these experiments started as population genomic surveys and thus a standard power analysis was not necessarily appropriate. However, below we discuss why we chose the numbers we did for each experiment:

1. *Population genomic survey*: We collected in the four Sky Island populations because they were areas that we and others had successfully collected in past years. Collections lasted for about two weeks with Dr. Hill driving between sites each day to collect. Previous studies also suggested that the frequency of DiNV infection would be high in these populations (Unckless 2001, our collections in 2016). So when deciding what to sequence, we attempted to get a balance from each population – shooting for 60-100 each. This number would more-or-less guarantee that we would obtain sequences for hundreds of infected individuals and allow robust population genomic inference. We also included 35 male individuals from a 2001 collection because they were available in a freezer and provided a useful comparison. Note that our confidence in our power to detect a signal in the GWAS was low and, in fact, the host-genome-based GWAS was likely underpowered (and is not the focus of the work presented here). The inherent noisiness of the wild collection, did not obscure the clear signal of our low and high haplotypes in the viral-genome-based GWAS. For the GWAS analysis, we used 254 infected flies and 1403 SNPs. It is difficult to estimate additive genetic variance, but other studies with other viruses find on the order of *h^2^*=0.3. This would yield power close to one (>0.99) with our parameters. Relaxing to *h^2^*=0.1 would give 0.32 and *h^2^*=0.2 would give 0.95.
2. *RNA-seq from wild caught-individuals and relation to viral genotype:* Again, we were mostly limited by our collections for this experiment so we used 40 infected and 40 uninfected individuals. Even with the noisiness of using wild-caught individuals, the use of 40 infected vs. 40 uninfected individuals is significantly more than a typical experiment, so we were confident in our ability to measure differences in gene expression. The secondary analysis – comparing expression in high- vs. low- type individuals was harder to predict *a priori* because we did not know which flies would be high and which would be low type. However, we recovered 26 high-type and 14 low-type, which again was reasonable to find differences in expression between types.
3. *Experimental infections in D. innubila:* We infected 30 individual male flies for each of 8 viral isolates (4 high-type and 4 low-type) for survival assays. For these samples we sequenced DNA from a portion of the homogenate to confirm viral type before infections. Again, we did not calculate power *a priori*, but these numbers are generally greater than what is standard in the field.
4. *Genome sequencing of D.* *falleni and D. azteca*: We again sequenced what was available, but made an attempt to bias toward infected individuals by screening for virus beforehand using PCR, since we were primarily interested in the high- vs. low- dynamics.

**Replicates**

- You should report how often each experiment was performed
- You should include a definition of biological versus technical replication
- The data obtained should be provided and sufficient information should be provided to indicate the number of independent biological and/or technical replicates
- If you encountered any outliers, you should describe how these were handled
- Criteria for exclusion/inclusion of data should be clearly stated
- High-throughput sequence data should be uploaded before submission, with a private link for reviewers provided (these are available from both GEO and ArrayExpress)

Please outline where this information can be found within the submission (e.g., sections or figure legends), or explain why this information doesn’t apply to your submission:

This information can be found in the materials and methods section of the manuscript.

Experimental infections:

For viral titer: We used 2-3 replicates per treatment (depending on the number of flies we were able to collect for infections at given times, and the number that survive to the given time point) per timepoint as this is the standard number, for each replicate we used 5 individual flies which we pooled for DNA isolation, though each fly was infected separately.

For survival, we infected 3 groups of 10 for each of eight viral genotypes or five dilutions. Each group was kept in a separate vial.

These experiments were conducted in one large block.

Sequencing data sets:

All sequencing data has been uploaded to the SRA (SRP187240) and processed data is available at DRYAD (https://datadryad.org/stash/share/wvfmDL39pdYrVUcgDFAfI33BOJu3KCJWuJyj-0M-qgA). The SRA data has not yet been made available to the public but can be if necessary.

**Statistical reporting**

- Statistical analysis methods should be described and justified
- Raw data should be presented in figures whenever informative to do so (typically when N per group is less than 10)
- For each experiment, you should identify the statistical tests used, exact values of N, definitions of center, methods of multiple test correction, and dispersion and precision measures (e.g., mean, median, SD, SEM, confidence intervals; and, for the major substantive results, a measure of effect size (e.g., Pearson's r, Cohen's d)
- Report exact p-values wherever possible alongside the summary statistics and 95% confidence intervals. These should be reported for all key questions and not only when the p-value is less than 0.05.

Please outline where this information can be found within the submission (e.g., sections or figure legends), or explain why this information doesn’t apply to your submission:

This information can be found throughout the manuscript, and is given in the text of the results, in some cases where more detail is required we have outlined the statistical tests and methods used in the methods. In all cases we have reported the p-value, the associated test used to find the p-value and the test statistic in the results when necessary.

We have presented the raw data and the distribution of the data whenever possible, and in cases with very small sample sizes (e.g. 2 to 3 replicates), have explained the reasoning behind the lack of replicates, mostly due to a lack of surviving flies to perform qPCR on.

(For large datasets, or papers with a very large number of statistical tests, you may upload a single table file with tests, Ns, etc., with reference to sections in the manuscript.)

**Group allocation**

- Indicate how samples were allocated into experimental groups (in the case of clinical studies, please specify allocation to treatment method); if randomization was used, please also state if restricted randomization was applied
- Indicate if masking was used during group allocation, data collection and/or data analysis

Please outline where this information can be found within the submission (e.g., sections or figure legends), or explain why this information doesn’t apply to your submission:

This is largely not applicable to the current study, but as mentioned above, we did pool 5 individuals for each qPCR data point. Pools of flies were chosen at random,grouping together 5 flies from the same vial of 10 flies.

**Additional data files (“source data”)**

- We encourage you to upload relevant additional data files, such as numerical data that are represented as a graph in a figure, or as a summary table
- Where provided, these should be in the most useful format, and they can be uploaded as “Source data” files linked to a main figure or table
- Include model definition files including the full list of parameters used
- Include code used for data analysis (e.g., R, MatLab)
- Avoid stating that data files are “available upon request”

Please indicate the figures or tables for which source data files have been provided:

We have uploaded all data used to generate figures and tables in the manuscript to data dyrad, with data available here: <https://datadryad.org/stash/share/wvfmDL39pdYrVUcgDFAfI33BOJu3KCJWuJyj-0M-qgA>

Sequencing data sets are available at NCBI under accessions: SRP187240
